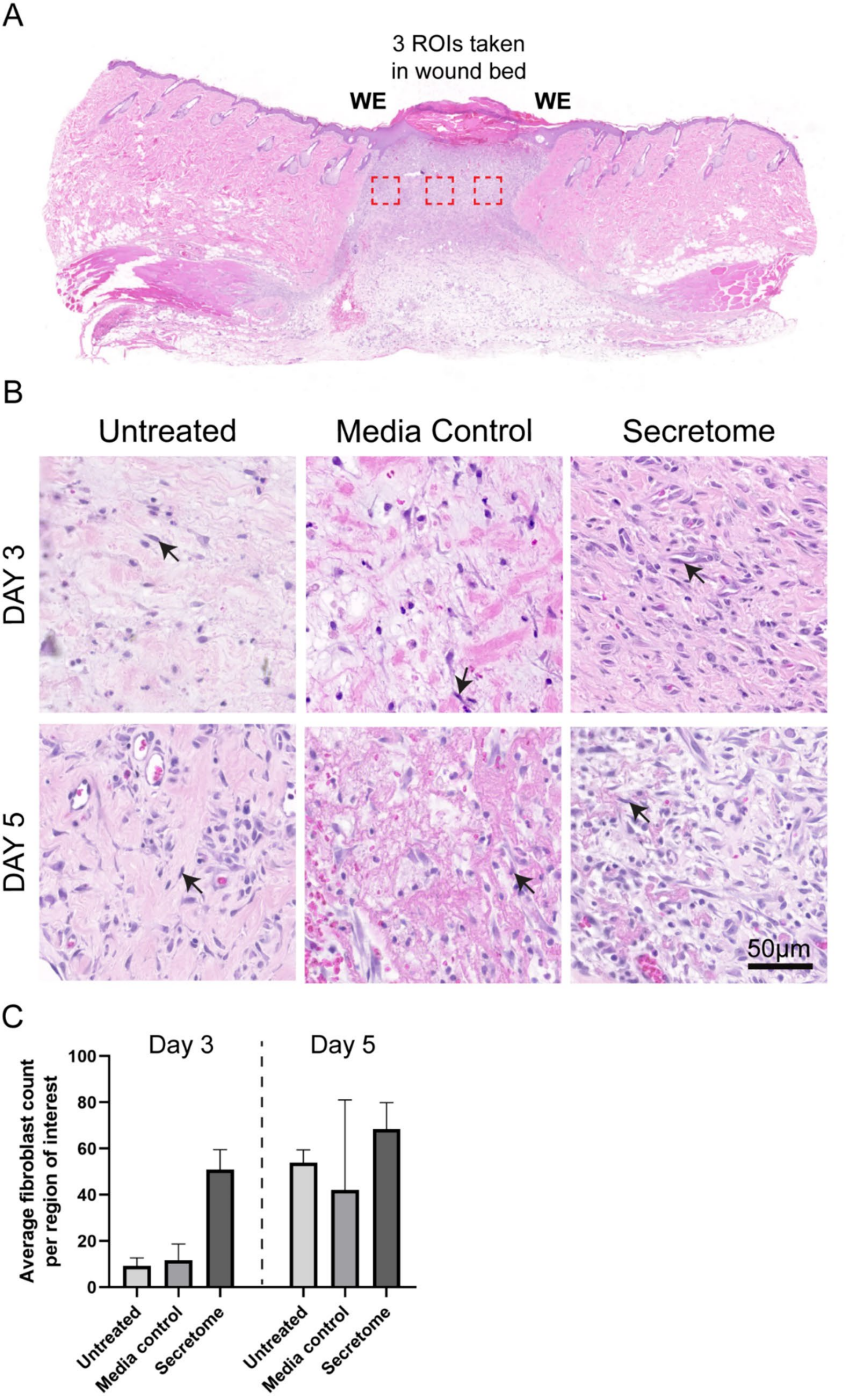# Correction to “Secretome From Prolonged High‐Density Human Wharton's Jelly Stem Cell Culture Accelerates Wound Healing in Both In Vitro and In Vivo Models”

**DOI:** 10.1111/iwj.70706

**Published:** 2025-06-08

Chin JS , Tan MLL , Lim PLK , et al. Secretome from prolonged high‐density human Wharton's jelly stem cell culture accelerates wound healing in both in vitro and in vivo models. Int Wound J. 2025; 22(5):e70033. 10.1111/iwj.70033 40320827 PMC12050407

Figure 8 is an erroneous duplicate of Figure 6. The legend for Figure 8 is correct, but the histology images and graph are wrong. The correct Figure 8 is provided below.

We apologize for this error.